# Supplementary material for: Are carbon emissions trading and green financial instruments synergistic? -Comprehensive quantitative research based on content analysis
Source: PLoS One. 2024 Mar 7;19(3):e0298601. doi: 10.1371/journal.pone.0298601 (PMC10919601; doi:10.1371/journal.pone.0298601)
Supplement: S1 Data — (ZIP) [file pone.0298601.s001.zip › Data/地级市Z_F工作报告.zip!/╕ú╜¿/─╧╞╜╩╨/└┤╫╘╣1⁄2╓┌║┼í╛┬φ┐╦╩2╛▌═°í┐_2014.docx]

各位代表：

　　现在，我代表南平市人民政府向大会报告政府工作，请予审议，并请市政协各位委员和其他列席人员提出意见。

　　一、2013年工作回顾

　　2013年，在省委、省政府和市委的正确领导下，我市各级政府认真学习贯彻党的十八大及十八届二中、三中全会和习近平总书记系列重要讲话精神，围绕创建全国绿色发展示范区目标，按照“发展要赶超、改革要争先、稳定要创优、环境要做美、民生要做好、作风要过硬”的要求，大力发展绿色经济、建设绿色城乡、倡导绿色消费、保护绿色生态，经济社会发展速度加快、效益提升、后劲增强，绿色发展成效进一步显现。

　　初步统计，全市生产总值1105.8亿元，增长11.2%；公共财政总收入106.7亿元，增长15.9%，地方公共财政收入71.6亿元，增长21%；全社会固定资产投资1214.5亿元，增长35.1%；外贸出口15.4亿美元，下降9%；验资口径实际利用外资1亿美元，增长20.2%；实际利用内资108亿元，增长15%；旅游接待总人数2106万人次，旅游总收入259亿元，分别增长14.5%、14.7%；城镇登记失业率3.4%；社会消费品零售总额400亿元，增长15.5%；居民消费价格总水平上涨2.6%；人口自然增长率6.5‰；城镇居民人均可支配收入24318元，增长9.4%；农民人均纯收入10031元，增长12.8%，顺利实现“311”目标。

　　（一）坚持集中集聚，主导产业更加凸显。坚持立龙头铸链条建集群，食品加工、旅游、机电制造千亿产业和生物五百亿产业行动计划顺利实施，明确市本级和各县（市、区）专业园区产业发展方向，推动“5+3”主导产业特色化、集聚化发展，规模以上工业增加值354.8亿元、增长14.5%。实施重点工业技改项目201项，圣农集团光泽扩建项目和浦城肉鸡加工厂部分建成投产，政和、松溪新建项目加快建设，福矛生产线二期等一批扩建项目投产，工业项目完成投资431.5亿元、增长39.9%，产值超亿元企业331家、净增74家。武夷新区高新技术产业园、荣华山新型轻纺园等专业园区建设加快推进，延平新城电缆城启动建设。商贸物流、电子商务等第三产业加快发展，“特色中国·武夷馆”等电商平台投入使用。新创福建省著名商标32件、地理标志证明商标3件、境外商标注册20件。武夷山自驾游营地等旅游项目相继建成，武夷山风景名胜区成为国家首批生态旅游示范区，新增国家级自然保护区1个、国家4A级旅游景区1个、3A级旅游景区2个，旅游接待能力有效提升。

　　（二）突破难点弱点，重点建设顺利实施。全力破解项目建设融资难、征迁难等问题和薄弱环节，“五大战役”全面超额完成年度目标，126个迎接高铁时代到来重点项目加快实施，城乡基础设施六项提升工程启动建设。成功发行武夷新区18亿元、高速公路公司二期15亿元债券和全市首支中小企业私募债券5000万元，新组建4家开发公司，市属国有集团进一步壮大，融资渠道有效拓展。创新征迁工作机制，重点建设用地得到保障。京福高铁南平段加快建设，南三龙铁路开工建设；京台高速公路南平段、邵光高速公路加快建设，延顺、南平联络线高速公路全线开工；武夷山机场国际候机楼竣工，新机场首选场址确定；建成南平中心城市交通枢纽，完成普通公路提升改造975公里；建成220千伏变电站1座、110千伏变电站7座。现代立体交通等重大基础设施更加完善，与沿海发达地区的时空距离大幅缩短。

　　（三）主动借势借力，招商引资富有成效。强化产业链招商、专业招商和以商招商，组织参加“6·18”、“9·8”等重大展会，成功举办“5·13”投洽会、第七届茶博会等重大经贸活动，开展10多场对外招商推介活动，推进与央企、民企、外企和省属企业项目对接，引进总投资2000万元以上合同项目876个、总投资2042亿元，项目开工率达70%。积极向上争取政策和项目资金支持，5个县（市、区）被新认定为原中央苏区县，闽北全境均为原中央苏区县；成功争取国家节能减排财政政策综合示范城市、循环经济示范城市、智慧城市试点政策；全年争取上级各类补助资金105亿元，增长16.2%，其中专项补助资金73亿元，增长23.7%，政策叠加效应逐步显现。

　　（四）突出绿化美化，城乡面貌明显改观。武夷新区开发建设全面推进，重点片区、重要节点以及各专项规划编制基本完成，南林核心区、将口片区、兴田片区、武夷高新技术产业园区等四大片区道路桥梁基础设施建设立体交叉推进，快速通道和轨道交通新岭至将口一期工程开工建设，建成3公里崇阳溪旅游景观带示范段，森林生态博物院等项目前期工作加快推进，南林、赤岸、芹口统建房等公建项目建设进展顺利。南平中心城市加快建设，延平新城建设全面展开，大家厂、湖尾城市综合体项目有序推进，滨江东路、闽江路全线贯通，完成高速公路南北互通口景观改造提升工程、八一路北段“白改黑”工程，朱熹路、九峰公园绿道一期工程基本建成，名仕园、雅阁2家五星级饭店加快建设。各县（市）加快实施城市建设“六个一”工程，相继完成街道改造、美食街建设、道路白改黑、高星级酒店、商贸综合体等一批项目；省级、市级小城镇改革试点工作顺利实施，5个村入选全国美丽乡村试点村，绿色城乡建设取得阶段性成效。

　　（五）着力增产增收，现代农业加快发展。加快推进农业产业化经营，培育省级以上农业产业化重点龙头企业50家、市级118家，农林牧渔业总产值423.4亿元，增长5.2%。“十个一批”现代农业示范项目、省级农民创业园和省级农民创业示范基地加快建设，完成2.5万亩生态茶园建设。推进土地流转54.2万亩，培育家庭农场115个、农民专业合作社767个。农业“五新”示范推广84项，农业机械化水平54%，新增设施农业面积1万亩。实施烟基工程和高标准农田建设11.3万亩，补充耕地2.5万亩。完成水利建设投资22亿元，新开工重点水利项目21个，水库除险加固75座，解决56.2万农村人口饮水安全问题。治理水土流失25万亩。实施农村路网建设与改造326公里、农村公路安保工程2137公里。5个省级扶贫开发重点县对接帮扶项目142个，落实资金14.3亿元。完成农村造福工程危房改造8350户、3.4万人，新安排的20个集中安置区基本建成。

　　（六）保护青山绿水，生态环境持续优化。生态市建设加快推进，新创建国家级生态乡镇55个，省级生态县（市）4个、生态乡镇17个。完成80个工业减排项目、90家畜禽养殖治理项目、31个闽江流域重点整治项目治理任务，节能减排年度目标全面实现。规范矿产开发秩序，开展矿山开采整治。推广清洁交通工具，全市配置液化天然气公交车150辆、双燃料环保动力出租车193辆。加强森林资源保护，造林绿化面积52万亩，建成福银、浦南高速公路森林生态景观示范段72片2002亩，完成205国道277公里绿色生态示范路建设，森林覆盖率提升至74.75%，生态环境质量继续保持全国全省前列。

　　（七）注重惠民利民，社会建设全面推进。为民办实事项目全面完成。新增城镇就业2.5万人，农村劳动力转移就业5.5万人。推进社会保障体系建设，完成城乡低保提标工作，全面实施临时救助制度，建立老年人高龄补贴制度。新建2个县级社会福利中心、10个社区综合服务站和24所乡镇敬老院。保障性安居工程开工6807套、基本建成10267套，超额完成年度任务。落实教育惠民政策，提前两年实现“双高普九”全覆盖，新扩建20所公办幼儿园，完成446所义务教育标准化学校创建，武夷学院通过本科教育合格评估。举办第六届科技成果交易会，强化知识产权工作，市本级和8个县（市、区）通过全国科技进步考核。人才强市战略深入实施，人才引进力度加大。新扩建4个县级图书馆，建成8个县级城区数字影院、37个群众性文化激情广场示范点。新增医疗机构床位1166张，全市公立基层医疗卫生机构和建制村卫生所全面实施国家基本药物制度。人口计生综合治理“三高”取得明显成效，计划生育工作整体水平有效提升。群众体育和竞技体育运动蓬勃开展，建成2个县级体育场馆。群众性精神文明创建活动持续深化，全面启动文明城市、卫生城市创建工作。妇女儿童、老龄和残疾人工作得到加强，社会福利和慈善事业稳步发展。“平安南平”建设深入推进，领导干部接访下访活动扎实开展，信访积案有效化解，社会保持安定稳定，综治考评名列全省第一。集中开展安全生产大检查和专项整治，道路交通安全综合整治“三年行动”和农村志愿消防队建设取得明显成效。援藏援疆援宁深入推进。民族宗教、外事侨务、气象防震、档案方志、老区库区等工作继续加强。军政军民团结巩固发展，市本级和6个县（市、区）获“福建省双拥模范城”称号。

　　（八）提升效能效率，政府自身建设得到加强。自觉接受人大和政协的监督，办理人大代表议案1件、建议133件和政协委员提案290件，满意率99.53%。广泛接受社会监督，虚心听取民主党派、工商联、无党派人士和社会各界的意见建议。推进“三级联动、重心下移”，联系服务群众机制不断健全。深化机关效能建设，精简审批事项，全面开展网上行政执法，推行“马上就办”，发展环境持续优化。严格落实“八项规定”，坚决反对“四风”，全市预算单位“三公”经费支出下降10.2%，廉政建设和行政监察、审计监督、纠风工作进一步加强。

　　各位代表，过去一年的成绩来之不易，这是省委、省政府和市委正确领导的结果，是全市人民齐心协力、团结奋斗和各方面大力支持的结果。我代表南平市人民政府，向全市人民，向全体人大代表、政协委员，向各民主党派、工商联、各人民团体、无党派人士和离退休老同志，向中央、省直驻南平机构和驻军、武警、公安民警，向对口帮扶地区，向关心支持南平发展的港澳台同胞、海外侨胞、国际友人，表示衷心的感谢！

　　在肯定成绩的同时，我们也清醒地看到发展还面临不少困难和问题，主要是：经济总量较小，产业发展实力不强，第三产业发展相对滞后；城乡规划建设管理水平有待提升，一些地方存在违法占地、违法建设现象，农村基础设施建设任务依然繁重；生态环境保护和节能减排压力较大；一些干部的思想观念、工作作风、执行力还不能适应推动科学发展、跨越发展的要求。对这些问题，我们将采取切实有效的措施，努力加以解决。

　　二、扎实做好2014年工作

　　今年政府工作的总体要求是：深入贯彻党的十八大及十八届二中、三中全会和习近平总书记系列重要讲话精神，按照省委九届十次全会、全省经济工作会议、市委四届六次全会的部署，围绕创建全国绿色发展示范区目标，坚持稳中求进、改革创新，全面迎接高铁时代到来，更加注重转方式调结构、兴产业强支撑、抓城乡优环境、惠民生促和谐，着力提高发展的质量和效益，加快建设美丽南平，全力推动南平科学发展跨越发展，努力实现“百姓富、生态美”有机统一。

　　今年经济社会发展的主要预期目标是：全市生产总值增长11%，公共财政总收入和地方公共财政收入均增长12%，全社会固定资产投资增长22%，外贸出口增长10%，验资口径实际利用外资增长10%，实际利用内资增长15%，旅游接待人数和收入均增长20%，城镇登记失业率控制在4.2%以内，社会消费品零售总额增长15%，居民消费价格总水平涨幅控制在3.5%左右，人口自然增长率控制在9.5‰以内，城镇居民人均可支配收入增长10%，农民人均纯收入增长11.5%，完成单位生产总值能耗、化学需氧量、二氧化硫、氨氮、氮氧化物等年度节能减排任务。

　　上述预期目标切合我市实际，体现市委四届六次全会提出的“三个必须”的要求，我们要坚定信心、主动作为，以世界的眼光、国际的标准、闽北的特色，提升发展理念，全面深化改革，积极实施深化“五大战役”重点行动计划，在产业发展、城乡建设、生态保护方面取得更大成效，推动南平加速追赶、跨越发展。

　　（一）深化项目带动战略，全面迎接高铁时代

　　推进重点项目建设。实施重点项目320个，总投资3229亿元，其中在建项目234个，年度计划投资373亿元，新开工项目123个。建立节能减排项目考评机制，实施5个总投资73亿元的节能减排示范工程。围绕创建全国绿色发展示范区目标，实施100个总投资1600亿元的绿色发展示范项目。做深做实项目前期，建立健全项目库，确保项目滚动发展并逐年增加。

　　加快完善基础设施。全面实施迎接高铁时代到来行动方案，大力推进与高铁配套衔接的交通、旅游、城建、物流等重点项目建设，确保与高铁同步建成投入使用。建成京福高铁南平段、向莆铁路南平连接线九峰段隧道，加快建设南三龙铁路，力争开工建设衢宁铁路及浦城连接线。加快建设京台高速公路南平段和邵光、延顺、南平联络线高速公路，开工建设顺邵高速公路。加快国省道干线和农村公路改造升级，建设和改造普通公路530公里，实施316国道生态示范路建设，建设武夷山东站等4个高铁换乘枢纽站和2个货运枢纽。加快中海油海西天然气管网二期工程南平段、华电邵武火电厂扩建等项目建设，新建、扩建220千伏变电站3座、110千伏变电站6座。

　　（二）培育壮大绿色产业，促进发展方式转变

　　提升产业发展实力。围绕“5+3”主导产业，继续实施食品加工、旅游、机电制造千亿产业和生物五百亿产业行动计划，加快圣农产业化扩建、太阳电缆异地技改等94个总投资529亿元重点工业项目建设，延伸产业链条，做大产业规模。加快编制大武夷旅游创新提升规划，实施刚泰武夷天堂文化旅游综合体、邵武瀑布林温泉二期工程等45个总投资850亿元旅游重点项目，加强旅游标准化、智能化和公共服务体系建设，推进文旅融合，整合县（市、区）旅游资源，联手打造精品路线，提升旅游承载力、吸引力和辐射力，推动高铁时代旅游发展。加快培育商贸物流业，推进城市商贸综合体建设，支持武夷山陆地港、圣农冷链物流等企业发展，各县（市、区）规划建设一个千亩以上的物流园区。扶持会展业发展，完善会展设施和服务网络。实施电子商务发展三年行动计划，加快建设武夷山紫阳古城电子商务集聚区，规划建设武夷新区、南平工业园区电子商务集聚区。积极推进金融产品创新，吸引更多金融机构进驻我市设点展业，加快村镇银行组建步伐，完善金融监管，促进金融产业健康发展。

　　实施龙头带动计划。深入梳理主导产业的龙头企业，优先配置土地、资金、用工等要素资源，大力扶持、培育和引进龙头，带动产业加快发展。扶持圣农、太阳电缆、赢创嘉联、元力活性炭、南孚、欧浦登、福矛、亚达、鑫富、武夷星、正山堂、武夷味精、青松股份、浦城正大、神农菇业、海源新材料、金柏夷锂电池等现有企业发展，鼓励通过上市、发债、引进战略投资者等方式实现增资扩产、迅速扩张。培育一批有规模、有市场、发展潜力大的企业，推动上下游企业整合重组，尽快成长为龙头企业。围绕产业链缺失环节，加快引进一批实力雄厚、带动性强的龙头企业。完善中小企业公共服务平台网络，健全企业融资担保体系，推动中小微企业加快发展。实施品牌带动战略，用好“武夷”商标，提升企业知名度和竞争力。搭建政银企、政校企合作平台，强化资金、人才等要素保障，更好地服务企业发展。

　　打造专业园区平台。修编园区产业规划，优化产业布局，促进产业集群化发展，形成各具特色的专业产业园。完善园区道路、供水管网、污水处理、电力、通信等基础设施，并结合城镇建设逐步强化园区商贸、文化、医疗等配套服务。健全园区管理体制，加快武夷新区高新技术产业园、南平工业园区、荣华山产业组团发展，推进电缆城、白炭黑林产化工园等循环经济园建设。

　　（三）夯实“三农”基层基础，提升农业现代化水平

　　大力发展现代农业。落实强农惠农富农政策，稳定粮食播种面积，确保粮食安全和重要农产品有效供给。实施现代农业绿色发展行动计划，加快建设连片设施农业基地，培育设施农业特色乡镇和专业村，提高种养综合生产能力和标准化生产水平，打造现代农业示范园。因地制宜推广种植丹桂、桔柚、锥栗、百合花、薏米、海鲜菇等地理标志产品和特色农产品，加快发展茶叶、烟叶、笋竹、油茶、食用菌、中药材等产业，打造10个区域特色农业。新植或补植无患子35万亩，大力引进龙头企业，促进产业发展。培育集种植、养殖、加工、休闲等为一体的林下经济，争创省级林下经济示范县。推进休闲农业和乡村旅游融合发展，加快一批山庄、茶庄、果庄、渔庄等“武夷品牌”休闲农庄和三星级以上乡村旅游经营单位建设。加强农业“五新”技术推广，加快智慧农业项目建设，提升农村信息化水平。开展农田土壤成份调查，鼓励推广使用有机肥，加强无公害、绿色和有机农产品认证，建立原产地可追溯制度和质量标识制度，确保农产品质量安全。

　　改善农村基础设施。推进光泽县茶富水库前期工作，实施松溪县虎洋水库等10个重大水利项目，完成15座小型病险水库除险加固工程。实施农村饮水安全工程，解决18万农村人口饮水安全问题。改造和建设农村公路320公里，实施农村公路安保工程1200公里，新增、更新农村客车101辆。

　　构建新型农业经营体系。规范提升农民合作社，发展家庭农场100家，成立绿发集团，促进农业龙头企业集聚发展，大力培养新型职业农民。加强基层农业公共服务机构能力建设，加快构建新型农业服务体系。稳定农村土地承包关系，放活土地经营权。建立县级土地流转服务平台试点，加快推进土地流转，实现适度规模经营。盘活村级集体资产资源，壮大村级集体经济。

　　加大扶贫攻坚力度。落实中央、省上扶贫开发政策，整合各部门扶贫开发资金，提高扶贫开发精准性，增强贫困地区自我发展能力。支持原中央苏区、革命老区、少数民族地区、偏远山区和库区发展，全面落实对口结对帮扶机制，推进山海协作共建产业园区，实施第四轮省级扶贫开发整村推进和老区村跨越发展工程，争取5个省级扶贫开发重点县财政收入、农民收入增幅高于全市平均水平，新增一批村财年收入10万元以上的建制村。

　　（四）全面建设武夷新区，拓展区域发展空间

　　优化新区功能布局。研究制定城市设计导则，科学合理布局南林核心区的市民中心、文化中心、科教中心、金融中心、商贸中心，高标准地推进建设，努力打造成为绿色发展生态文明建设的样板，形成资金、项目、人才等要素汇集的“洼地”。推动武夷山、建阳加快融入武夷新区，做好城市建筑风格风貌、市政公用设施、产业项目布局等方面的对接，促进同城化发展。

　　加快新区项目建设。全面推进武夷新区快速通道和轨道交通、崇阳溪旅游景观带、森林生态博物院、古汉城遗址公园等重点项目建设，加快博物馆、科技孵化大楼、核心区四所学校、森林公园等项目建设，启动浦南高速公路南林段西移改线、渡头大桥、崇阳溪两岸、商业街、风情街等路桥工程建设，进一步完善核心区城市骨干路网，迅速拉开新区城市框架。

　　促进新区产业集聚。坚持工业、商贸物流、旅游养生、文化创意产业协同发展，吸引总部经济和现代服务业进驻新区，推进与沿海发达地区共建产业园区。培育做大新材料新能源产业。加快引进食品加工、生物医药、机械电子等产业龙头项目，促成万达集团、复星集团等项目尽快开工建设，提升新区产业发展水平。

　　（五）做精做美绿色城乡，加快打造旅游城市

　　提升城乡规划和管理水平。坚持“绿色、特色、现代”理念，明确新城区发展方向，完善旧城区功能，突出“武夷风格”和闽北建筑风格特色，抓好城乡综合交通、风貌景观、公共要素配套等专项规划设计，加快建设旅游城市。严格城乡规划管理，加强城乡环境综合治理，大力打击“两违”和违章占道停车、乱摆摊经营等现象，提高城市品位。

　　积极开展新型城镇化试点。出台实施城镇化发展规划，加快推进绿色城市、特色城镇和美丽乡村建设，构建城乡协调发展的新型城镇化格局。扎实推进光泽县“生态食品城”、邵武市产城融合试点，加快省级、市级小城镇改革试点镇建设，积极引导偏远山区群众向中心镇和产业园区、县城周边集中集聚，有序推进农业转移人口市民化，提高新型城镇化水平。

　　建设山水园林城市。实施宜居环境建设行动计划，延伸拓展“点线面”攻坚，深入推进“五千工程”，提升城区“三边三节点”，保护城市自然生态和历史文脉。南平中心城市同步推进旧城改造提升和延平新城建设，建成水东新大桥、夏道大桥、滨江路跨线桥、朱熹路扩建及景观工程、城市二水源工程，加快建设水南新大桥，完成横排路、紫芝巷等12条市区道路及街巷改造，开工建设八仙等4个城市社区公园。持续推进各县（市、区）建设或改造提升一个完整社区、一条美食街、一个游客集散中心、一个主题文化公园、一个城市商贸综合体、一个专业产业园等“六个一”工程，力争年内取得明显成效。

　　加快打造美丽乡村。实施百个美丽乡村示范、千公里生态绿廊、万人保洁的“百千万”工程，制定农村住宅用地指导意见和建设导则，组建民间建筑工匠人才库，引导农民按照宜疏不宜密和两层半、坡屋顶的要求建设新房，弘扬闽北农村建筑特色，改善农村人居环境，展示山水田园风光。

　　（六）强化招商用好政策，增强经济发展后劲

　　加大招商选资力度。深入推进“三维”项目对接，突出重点产业招商、借助招商平台招商、利用地缘关系招商，大力实施“回归工程”，办好“5·13”投洽会和“11·16”茶博会，开展专场招商推介活动，引进一批大项目、好项目。落实招商优惠政策，强化重大项目跟踪服务，做好项目用地报批、征迁工作，切实改变项目等地状况，促成项目尽快落地投产。

　　扩大对外经贸合作。积极参与闽东北经济协作区、闽浙赣皖九方经济区和泛珠三角经贸合作洽谈，推动建立更加紧密的区域合作机制。发挥南平旅港、澳门南平联谊会和闽北海外社团的作用，深化与港澳台地区经贸文化交流合作，加强与国外友城沟通联系，拓展对外开放领域。鼓励企业参加各类展会，扶持重点出口企业做大做强，拓展国际市场。

　　积极发挥政策效应。用足用活国家节能减排、循环经济示范城市和低碳城市、智慧城市试点以及苏区老区库区、扶贫开发等政策，整合各方面资源，组建政策对接和项目策划工作班子，策划实施一批大项目。继续从全国绿色发展示范区、武夷山生物多样性以及生态补偿等方面去深入谋划，争取上级更多政策支持。

　　（七）加强生态文明建设，增创绿色发展优势

　　建设生态文明城市。完善生态市建设机制，严把新上项目产业政策、资源消耗和环境保护审批关，争取8个县（市）通过国家级生态县技术评估、2个县（区）创建省级生态县。持续推进文明城市建设，邵武、武夷山争创全国文明城市，其他县（市、区）争创省级文明县城。

　　加强生态资源保护。推进水资源综合管理系统建设，完成20万亩水土流失治理任务。开展矿山生态环境恢复和“矿山复绿行动”。严格耕地保护，加强土地整理复垦开发，促进节约集约用地。加强森林资源保护，严厉打击盗伐森林行为。加快“四绿”工程建设，植树造林30.3万亩，不断改善林分结构。落实生态补偿机制和管护机制，加大自然保护区和生态公益林的保护力度。启动实施316国道和30条农村公路生态示范样板路建设工程，推进福银高速公路邵武和平段森林生态景观通道和武夷新区环城一重山森林生态景观提升。

　　抓好环境综合治理。继续开展闽江流域水环境综合整治、农村环境连片整治，推进饮用水源保护、养殖污染整治、工业污染整治、城乡垃圾污水整治。深入开展整治违法排污企业保障群众健康环保专项行动。推动机动车污染防治，开展大气和重金属污染防治，新增PM2.5、一氧化碳、臭氧监测，提高环境监管能力。实施强制性清洁生产，完成59家企业审核验收工作。

　　（八）强化社会公共服务，切实保障改善民生

　　做好就业和社会保障工作。落实就业再就业政策，完善公共就业创业服务体系，新增城镇就业2.3万人，转移农村劳动力5.2万人。完善多层次住房供应体系，建设保障性住房4429套。巩固提高新农合参合率，城乡居民基本医保的财政补助标准提高到每人每年320元。全面实施城乡居民养老保险制度一体化，扩大参保缴费覆盖面，稳妥推进城镇居民大额医疗费用商业补充保险试点。加快县级社会福利中心和乡镇敬老院建设，推动养老服务业发展。建立健全农村留守儿童、妇女、老年人、失独家庭关爱服务体系，积极发展慈善和残疾人事业。

　　坚持教育优先发展。实施新一轮学前教育发展三年行动计划，新增10所公办幼儿园。建成18所义务教育标准化学校，延平、邵武、武夷山、建阳通过国家义务教育基本均衡验收。继续推进城区中小学扩容提质，鼓励名校办分校、老校带新校、强校扶弱校。构建以就业为导向的现代职业教育体系，支持福建林业职业技术学院、闽北职业技术学院提高办学水平，完成闽北卫校新校区搬迁。支持武夷学院申报专业硕士点和福建农林大学南平校区扩大办学规模。扶持特殊教育，推动民办教育规范发展。

　　提高卫生计生服务水平。全面深化医疗卫生、人口计生体制机制改革，推进公立医院改革，探索组建医疗集团，鼓励社会资本办医。完善基本药物制度，促进基本药物优先合理配备使用。优化医疗资源规划布局，加快南平市第一医院综合住院病房大楼等项目建设，提升县乡医疗机构服务能力。推进卫生信息化建设，完善“健康云”服务平台。坚持人口计划生育基本国策，建立和完善稳定低生育水平的长效机制，加大出生人口性别比综合治理力度，提高出生人口素质。

　　加快科技和人才创新体系建设。支持企业与高校、科研机构共建研发中心、实验室等科技创新载体，鼓励多元化投资建设专业化科技孵化器。办好科技成果交易会，加快邵武、武夷山省级农业科技园区和武夷新区科技创意产业园建设。加强知识产权保护，支持申报专利。创新人才工作机制，加大人才工作力度，鼓励和吸引各类人才到我市创业和发展。

　　促进公共文化繁荣发展。加强社会主义核心价值体系建设，提高精神文明创建实效。继续实施文化惠民工程，完善城乡四级公共文化服务体系。加强文化遗产的传承和保护，做好历史文化资源和文物保护，加快建设一批古镇古村文化旅游项目，打造朱子文化、武夷茶道、闽越文化等特色文化品牌。办好第十一届闽浙赣皖四省四市民间艺术节。探索组建文化集团，建设文化创意产业园区。广泛开展全民健身运动，不断提高群众身体素质。

　　推进社会治理创新。深化拓展“南平机制”，加快构建农技服务、农村流通和综治服务平台。严格执行重大决策社会稳定风险评估机制，继续推进城乡社区网格化服务管理，推广农村社会治理“1+3”新模式，完善矛盾纠纷多元化解、健全医患纠纷“南平解法”长效机制，深入实施信访“路线图”，积极构建“大调解”工作体系。深化“平安南平”建设，健全社会治安防控体系，依法惩治各类违法犯罪活动。深化法制宣传教育，完善法律援助制度。健全社区矫正制度，强化安置帮教工作。实施安全发展战略，建立重点行业领域隐患排查治理和安全预防控制体系，继续开展道路交通安全综合整治。加强食品药品安全监管，持续治理“餐桌污染”，建设“食品放心工程”。落实党的民族政策和宗教工作基本方针。支持工会、共青团、妇联等群团组织积极开展工作。深化“双拥”共建，支持驻军部队建设，强化国防教育、国防动员、国防后备力量和人民防空建设。

　　三、全面深化改革，为绿色发展提供机制体制保障

　　（一）解放思想，致力先行先试。以敢闯的锐气和会闯的睿智，大胆探索，着力先行，全面深化改革，努力在创优发展环境和创新农业经营方式、旅游发展体制机制、社会治理体制机制等重点领域和关键环节的改革上取得突破，在创新生态文明建设体制机制上争当全国全省改革的“排头兵”，推动南平在新的发展起点上赢得先机。

　　（二）简政放权，激发市场活力。完成市县两级政府机构改革，稳妥推进事业单位分类改革，加快政府职能转变。推进食品药品监管体制改革，理顺监管职责。深化行政审批制度改革，重点推动工业园区项目并联审批和代办制，推进工商登记制度改革，营造宽进严管的市场准入环境。完成第三次全国经济普查工作。加强国有资产监管，推进国有企业改革，完善现代企业制度。落实支持民营经济发展的政策措施。推进资源要素市场化配置，深化土地使用制度改革，探索山林资源流转改革，推进资源性产品价格改革，建立统一规范的公共资源交易平台。

　　（三）改进作风，优化服务环境。贯彻落实中央“八项规定”精神，严格执行党政机关厉行节约反对浪费、公务接待管理和效能建设等工作条例。扎实开展党的群众路线教育实践活动，深入推进“三级联动、重心下移”联系服务群众的工作机制，办好为民实事。全面推行一线工作法，以“踏石留印、抓铁有痕”和钉钉子的精神抓好落实。强化政府绩效管理，完善“马上就办、办就办好”机制，精简检查评比活动，营造良好的发展环境。

　　（四）依法行政，建设法治政府。坚持依法治市，自觉接受人大、政协监督，接受舆论和群众监督，加强经济责任审计，强化行政执法监督，规范行政权力运行。认真落实“一岗双责”，严格执行党风廉政建设的各项规定，健全惩治和预防腐败体系，坚决查处违法违纪案件。深化财税体制改革，强化预算约束，做好“三公”经费预决算公开试点工作。坚持用制度管权管事管人，令行禁止、克己奉公，以清正廉洁的形象取信于民。

　　各位代表，让我们紧密团结在以习近平同志为总书记的党中央周围，在省委、省政府和市委的坚强领导下，紧紧依靠全市人民，坚持绿色发展走新路，锐意进取，攻坚克难，努力实现“百姓富、生态美”有机统一，为加快推进南平科学发展跨越发展、全面建成小康社会而努力奋斗！
